# Retraction: Novel thiosemicarbazone UV-vis chemosensor for dual pH-orthogonal detection of Fe^3+^ and Ag^+^

**DOI:** 10.1039/d6ra90072k

**Published:** 2026-07-22

**Authors:** Sherin A. M. Ali, Mostafa E. Salem, Ahmed Z. Ibrahim, Mostafa A. A. Mahmoud, Mohamed Abdel-Megid, Belal H. M. Hussein

**Affiliations:** a Department of Mechanics, Faculty of Engineering, Suez Canal University Ismailia 41522 Egypt; b Chemistry Department, College of Science, Imam Mohammad Ibn Saud Islamic University (IMSIU) Riyadh 11623 Saudi Arabia; c Chemistry Department, Faculty of Science, Suez Canal University Ismailia 41522 Egypt a.zaki_89@yahoo.com

## Abstract

Retraction of ‘Novel thiosemicarbazone UV-vis chemosensor for dual pH-orthogonal detection of Fe^3+^ and Ag^+^’ by Sherin A. M. Ali *et al.*, *RSC Adv.*, 2026, **16**, 3850–3867, https://doi.org/10.1039/d5ra07794j.

The Royal Society of Chemistry, with the agreement of the authors, hereby wholly retracts this *RSC Advances* article due to inaccuracies within the reference list. The authors have identified errors in a number of references in the list, and the extent of these errors is too large for a Correction. Therefore, we are retracting this article. No concerns have been raised regarding the experimental data, figures, results, or conclusions of the article.

The authors were informed about the retraction of the article. Ahmed Z Ibrahim and Belal H. M. Hussein have agreed with the decision, the other authors have not responded.

Signed: Ahmed Z. Ibrahim and Belal H. M. Hussein

Date: 10th July 2026

Retraction endorsed by Laura Fisher, Executive Editor, *RSC Advances*

